# Sleep education with self-help treatment and sleep health promotion for mental and physical wellness in Japan

**DOI:** 10.1007/s41105-015-0018-6

**Published:** 2015-12-09

**Authors:** Hideki Tanaka, Norihisa Tamura

**Affiliations:** Department of Psychology, Faculty of Psychology, Hiroshima International University, 555-36, Kurose-Gakuendai, Higashi-Hiroshima city, 739-2631 Hiroshima Japan; Department of Somnology, Tokyo Medical University, Tokyo, Japan

**Keywords:** Self-help treatment, Sleep education, Goal setting strategy, School, Elderly

## Abstract

The purpose of this article was to provide an overview of the effects of the sleep education with self-help treatment for student, teacher, and local resident and sleep health promotion for mental and physical wellness for elderly with actual examples of public health from the community and schools. Sleep education with self-help treatment in schools revealed that delayed or irregular sleep/wake patterns were significantly improved. Also, it was effective for improving sleep-onset latency, sleep satisfaction, mood during the morning, and daytime sleepiness. The strategy of this sleep education included the acquisition of the correct knowledge about sleep and the sleep-related behaviors that are important for improving sleep. Sleep health promotion that included short naps and exercise in the evening (Sleep health class) was effective in promoting sleep and mental health with elderly people. The interventions demonstrated that the proper awakening maintenance, keeping proper arousal level during the evening was effective in improving sleep quality. Furthermore, sleep management that included sleep education and cognitive-behavioral interventions improved sleep-related habits and the quality of sleep. In this study, a sleep educational program using minimal cognitive-behavioral modification techniques was developed. Mental and physical health was also improved with better sleep in the elderly. These results suggest that sleep health promotion is effective for mental and physical wellness for the elderly.

## Introduction

The Ministry of Health, Labor and Welfare in Japan proposed a plan called ‘‘Health Japan 21,’’ which adopted sleep as one of the specific living habits needing improvement. Many recent surveys in Japan have reported that one in three Japanese elderly individuals and one in five Japanese adults and students suffer from insomnia. Insomnia is becoming a serious social problem; insomnia is listed as one of the refractory diseases of the 21st century. It has been observed that today’s adolescents and children have nocturnal lifestyles and sleep for few hours.

Behavioral and educational interventions for healthy sleep are very important for ensuring proper sleep and regular lifestyle habits. There is increasing evidence that such non-pharmacological approaches produce reliable and durable sleep improvements. Comprehensive treatment is likely to include combinations of sleep hygiene education [1], including the regularization of the sleep/wake pattern across the week [2]; early morning bright light exposure [3]; stimulus control therapy [4]; and cognitive therapy to address unhelpful sleep beliefs [5]. Thus, behavioral and cognitive strategies have extensive empirical support for adults [6]. The benefits of sleep education with self-help treatment have been demonstrated for a variety of sleep problems and daytime consequences [7]. Sleep education with self-help treatment for students in high school revealed that delayed or irregular sleep/wake patterns were significantly improved [8].

Also, it was effective for improving sleep-onset latency, sleep satisfaction, mood during the morning, and daytime sleepiness. The strategy of this sleep education included the acquisition of the correct knowledge about sleep and the sleep-related behaviors that are important for improving sleep.

The purpose of this article was to provide an overview of the effects of the sleep education with self-help treatment for student, teacher, and local resident and sleep health promotion for mental and physical wellness for elderly with actual examples of public health from the community and schools.

### Sleep education with self-help treatment for elementary schoolchild in Japan

We examined the effects of sleep education with self-help treatment in elementary school children presenting with a nocturnal lifestyle [9]. One-hundred and forty-eight children from two schools participated in this study. Schools provided one group for sleep education and a second group as controls. Ethics approval for the research project was obtained from the Hiroshima International University Medical Research Ethics Committee. Also, the research was carried out at an elementary school where the school principal was requiring sleep education as a part of efforts to promote the improvement of lifestyle habits for the children. In the interests of ethical niceties towards the control group, we carried out the sleep education for children’s parents and teachers after the research.

The sleep education with self-help treatment ran for 2 weeks in June. The sleep educational materials were made according to grade level and the sleep class carried out for 45 min by each grade level. The children were asked to fill out the sleep ○ × quiz (Table 1) before being provided with the knowledge about sleep and then the sleep instructor lectured them regarding sleep hygiene (the knowledge about both the importance of sleep health and of sleep improvement) for 20 min. Next, the children were asked to check their own lifestyle habits and sleep states for 15 min using both the checklist of lifestyle rhythms (Table 2 left items, Cronbach *α* = 0.934) and the sleep and daytime functioning questionnaire. Also, when the children checked their own lifestyle habits, they were asked to select a target behavior to set a goal for change and to practice the selected target behavior for 2 weeks using a sleep diary. The instructor showed them how to fill out the diary in about 10 min and asked them to monitor the selected target behavior for 2 weeks. Finally, to check the effect of the sleep class, the sleep ○ × quiz was carried out again after the class. Also, children selected one practice effective for improving sleep, and the selected target behavior was monitored by them for 2 weeks. After 2 weeks, the children were asked to answer the sleep ○ × quiz, the checklist of lifestyle rhythms, and the sleep and daytime functioning questionnaire again.

**Table 1:** Sleep knowledge and checklist of sleep-promoting behaviors for primary school children

Sleep knowledge for primary school children		
Sleep knowledge quiz	Response	Correct answer
Q1. Are going early to bed, early rising and having breakfast good for the health?	( )	○
Q2. Are having breakfast and defecating every morning energy sources?	( )	○
Q3. Does insufficient sleep influence the mistake or an injury?	( )	○
Q4. Does insufficient sleep decrease brain activity?	( )	○
Q5. Does insufficient sleep cause weight gain?	( )	○
Q6. Does the human body have the rhythms or the chronometer?	( )	○
Q7. Is it better to expose oneself to sunlight in the morning?	( )	○
Q8. If you get sleepy just after coming back home, is it better to take a nap?	( )	×
Q9. When you feel having insufficient sleep, is it better to oversleep until afternoon on weekends?	( )	×
Q10. Is it good to refrain from going to the brightly lit places, such as a convenience store, just before sleep time?	( )	○

**Table 2:** Comparison of sleep-related behaviors between the sleep education and the control groups

Sleep-related behavior		Sleep education group (*n* = 72)				Control group (*n* = 76)				Between group
		○	△	×	*χ* ^2^	○	△	×	*χ* ^2^	*χ* ^2^
1. Getting up every morning approximately at a fixed time	Pretreatment	38.0	53.5	8.5	8.25**	43.4	46.1	10.5	1.04	0.84
	Post-treatment	63.4	32.4	4.2		35.5	51.3	13.2		12.24***
2. Exposing oneself to sunlight in the morning	Pretreatment	41.4	37.1	21.4	8.12*	40.8	42.1	17.1	0.48	0.59
	Post-treatment	57.1	37.1	5.7		35.5	44.7	19.7		9.73**
3. Having breakfast every morning	Pretreatment	91.5	7.0	1.4	0.74	84.0	13.3	2.7	0.11	1.71
	Post-treatment	94.4	5.6	0.0		85.3	8.0	6.7		4.78*
4. Not taking a nap after coming home	Pretreatment	76.8	17.4	5.8	2.80^†^	73.3	13.3	13.3	2.00	2.55
	Post-treatment	88.4	8.7	2.9		69.3	21.3	9.3		7.13**
5. Refraining from sleeping in the morning of holiday	Pretreatment	31.0	39.4	29.6	0.56	36.1	37.5	26.4	0.29	0.45
	Post-treatment	36.6	38.0	25.4		34.7	41.7	23.6		0.20
6. Not going out to brightly lit places, such as convenience store, before sleep	Pretreatment	77.5	14.1	8.5	3.69^†^	75.0	11.8	13.2	3.47^†^	0.92
	Post-treatment	87.3	11.3	1.4		86.8	6.6	6.6		0.52
7. Refraining from watching television or videos before sleep	Pretreatment	22.5	33.8	43.7	9.36**	25.0	43.4	31.6	0.84	2.40
	Post-treatment	46.5	26.8	26.8		26.3	48.7	25.0		8.82**
8. Refraining from playing video game before sleep	Pretreatment	60.6	19.7	19.7	4.98^†^	61.3	24.0	14.7	0.44	0.85
	Post-treatment	60.6	31.0	8.5		60.0	28.0	12.0		0.56
9. Going to bed every night approximately at a fixed time	Pretreatment	35.7	45.7	18.6	1.66	36.8	48.7	14.5	0.56	0.45
	Post-treatment	47.1	38.6	14.3		39.5	50.0	10.5		1.98
10. Exercising every day, or playing sport	Pretreatment	62.0	32.4	5.6	0.18	51.3	35.5	13.2	0.26	3.03
	Post-treatment	64.8	31.0	4.2		48.7	39.5	11.8		5.04^†^

***p < 0.001; **p < 0.01; *p < 0.05; ^†^ *p* < 0.1

After the sleep class, the total number of correct answers to sleep-related items in the sleep education group improved from pre-treatment to after the class and was maintained after 2 weeks. After the treatment, participants in the sleep education group showed significant improvements in bedtime and sleep duration. Moreover, their poor sleep and irritability significantly improved.

Some sleep-related behaviors associated with being efficient for sleep showed a significant increase. Ten items of sleep-related behaviors are shown in Table 2 in each group for two assessment phases. The ratio of participants in the sleep-education group that “get up every morning approximately at the decided time” was 38 % before the treatment compared with 63.4 % just after the treatment. After the treatment, the ratio of participants who had a regular rising time every morning increased in the sleep-education group compared with the control group (*P* = 0.001). In terms of “refraining from watching television or videos before sleep”, the ratio of participants was improved significantly in the sleep education group from pre- (22.5 %) to post-treatment (46.5 %). Furthermore, the sleep-related behaviors, such as “not taking a nap after coming home”, trended toward becoming more increased in the sleep education group post-treatment in comparison with pre-treatment.

### Relationship among sleep-related behaviors, bedtime, sleep duration, and mood in the morning

Based on the multiple regression analysis, bedtime and sleep duration were improved through the modification of three sleep-related behaviors including getting up every morning approximately at the decided time, refraining from watching television or videos before sleep, and not taking a nap after coming home. Furthermore, an improvement in bedtime and sleep duration is effective in alleviating irritability. These results suggest that sleep education with self-help treatment can be useful in improving nocturnal lifestyle, poor sleep, and irritability in elementary school children.

Recently, sleep education program has been conducted for sleep problems in adolescents. This program has included knowledge about sleep importance and proper sleep hygiene, and method to change sleep patterns [10], and has consisted of four 50-min classes across a 4-week period [11, 12]. Some study reviews suggest that sleep education program is effective in enhancing adolescent knowledge about sleep, but show less consistent success in improving behavioral sleep outcomes such as daytime sleepiness, sleep duration or sleep-promoting behavior [10, 13]. As method to resolve this problem, it is considered to be effective for assessing sleep-promoting behaviors individually by using a checklist which contained sleep-promoting behaviors. This method includes setting a target-based on their responses to the checklist, practicing and monitoring the target behavior by using sleep log. Sleep education program with self-help treatment using the checklist of sleep-promoting behaviors has already conducted in school students and elderly, which improved their sleep problems such as late bedtime and insufficient sleep [8, 9, 14].

### Sleep education for Junior high school students in Japan

Subjective insufficient sleep and delayed sleep-wake pattern have been known as the primary causes for daytime sleepiness in adolescents. Subjective insufficient sleep is attributable to short sleep duration due to late bedtime on school day [15] and is more frequently observed in the Japanese adolescents [16]. As for delayed sleep-wake pattern, it develops as a discrepancy in sleep-wake rhythm on school day and weekend, which may cause a delay shift of the biological clock and a tendency for higher rates of daytime sleepiness [17, 18]. Consequently, adolescents’ circadian rhythm might become difficult to synchronize with their school schedule and could have a greater risk of lowered daytime functioning.

Recently, our study [19] developed a sleep educational program using a checklist of sleep-promoting behavior which enables students to assess their sleep-promoting behaviors and to set a target behavior based on their responses, and evaluated its effect on sleep-promoting behaviors, sleep habits, and daytime sleepiness in adolescents.

Two-hundred and twenty-nine students at the first grade of two junior high schools were enrolled in this study: the sleep education group (*n* = 118) and the wait-list group (*n* = 111). The protocol of the current study was reviewed and approved by the Research Ethics Committee of Hiroshima International University. The present study was conducted at schools obtained consent from principals and class teachers. Then we provided study information with a consent form to the students through their classroom teacher and asked for their cooperation. Sleep education was carried out for 2 weeks and administered by a qualified sleep instructor. Class was conducted for each 50 min. We asked the students to fill out the sleep knowledge quiz (Table 3, upper) before being provided with the knowledge. Then the students were provided with information about proper sleep hygiene and sleep-promoting behaviors for 25 min using a Microsoft Power Point format. The contents of sleep hygiene included psychoeducation to raise students’ awareness of their sleep health and to motivate students towards practicing their sleep-promoting behaviors (Table 3, lower), in addition to correct answers of the sleep knowledge quiz. Following the sleep education, the students were asked to check their own sleep pattern and daytime functioning and to assess sleep-promoting behaviors for 25 min. Based on the response (△ but can practice) to checklist of sleep-promoting behaviors, the students were asked to practice one sleep-promoting behavior set as their goal for two weeks.

**Table 3:** Sleep knowledge and checklist of sleep-promoting behaviors for junior high school students

Sleep knowledge for junior high school students		
Sleep knowledge quiz	Response	Correct answer
Q1. Is the grade of obesity associated with sleep length?	( )	○
Q2. Is the cycle length of human body rhythm not just 24 hours?	( )	○
Q3. When you wake-up in the morning, is it better to keep the curtain closed?	( )	×
Q4. If you get sleepy just after coming back home, is it better to take a nap?	( )	×
Q5. When you feel having insufficient sleep, is it better to oversleep until afternoon on weekends?	( )	×
Q6. Does body temperature rise during nocturnal sleep time?	( )	×
Q7. Does use of a mobile phone in the bedroom bring good sleep?	( )	×
Q8. Does a tepid bath bring good sleep?	( )	○
Q9. If you have difficulty falling asleep at night, is it better to keep lying on bed until you fall asleep?	( )	×
Q10. Is it better to refrain from going to brightly lit places, such as a convenience store, just before sleep time?	( )	○

Checklist of sleep-promoting behaviors for junior high school students			
Sleep-promoting behaviors	Self-assessment		
1. Getting up at almost fixed time every morning	○	△	×
2. Exposing oneself to sunlight in the morning	○	△	×
3. Having breakfast every morning	○	△	×
4. Not taking a nap just after coming back home	○	△	×
5. Avoiding caffeinated drinks, such as tea or coffee, after dinner	○	△	×
6. Avoiding snacks late in the evening	○	△	×
7. Taking a tepid bath relaxedly 2 hrs before bedtime	○	△	×
8. Going to bed by 12:00 am at the least every night	○	△	×
9. Trying to rest the brain and mind before sleep	○	△	×
10. Keeping the difference of wake-up time of weekdays and holiday within 2 hour	○	△	×

○ practice, × do not practice, and △ but can practice

At the post-education periods, the sleep education group showed significant improvement of knowledge about sleep hygiene and the sleep-promoting behaviors. Their bedtime on both weekday and weekend, sleep onset latency, total sleep time on weekday, daytime sleepiness, and fatigue were also improved in the sleep education group. In contrast, no significant improvement of these variables was observed in the wait-list group other than shortened total sleep time on weekend. The results suggest that the newly developed sleep education program is effective for improving adolescents’ sleep-promoting behaviors, sleep habits, and daytime function.

### Sleep education with self-help treatment for junior high school students

The pilot study of sleep education aimed to verify a sleep educational program with self-help treatment using a checklist of sleep-promoting behaviors in adolescents [8]. We examined the effects of sleep education using short-term cognitive-behavioral method (knowledge and self-help treatment) for junior high school students. Furthermore, we examined effective behaviors that ensure sleep and prevent late night lifestyles. Sleep education using a self-help treatment was carried out for 10 days with 318 (ages 13, 14, and 15) participating students. Sleep instructors lectured the students regarding sleep hygiene using text and provide them with 10 questions relating to sleep before and after a lecture. In this program, the instructor asked each student to check his or her own life-habits, to select one target behavior, and keep a sleep diary for 10 days.

As a result of the lecture for the junior high school students, their knowledge of sleep significantly improved [20]. After 10 days of the sleep education by using self-help treatment, sleep habits, and delayed or irregular sleep-wake patterns significantly improved, and the students reported earlier bed times on school nights. The difference between weekday and holiday bedtimes shortened, significantly. Sleep latency, sleep satisfaction, mood during the morning, and sleepiness during the day significantly improved. These results show that the treatment was effective for preventing a night-type lifestyle and for reducing irregular sleep–wake patterns.

Furthermore, we examined a probability both of bed time advance with improvement for lifestyle and of improvement for each lifestyle with prevention of taking a nap just after coming back home in the evening. The probability analyses showed that having breakfast every morning and the practice of not taking a nap just after coming back home are effective for preventing late bedtime or irregular sleep–wake patterns. Furthermore, not taking a nap just after coming back home in the evening is related with exposing to sunlight in the morning, and trying to rest the brain and mind before sleep.

### Effects of sleep management for school nurses in elementary school and junior high school

The present study examined whether sleep education and self-help treatment by using sleep daily would improve the lifestyle-habits, sleep, mood of the morning, and volition of twenty-two school nurses in elementary school and junior high school [21]. After sleep education was performed, school nurses were divided into the only educational group (*n* = 11) and the self-help treatment group (*n* = 11). Sleep education were provided with information about proper sleep hygiene and sleep-promoting behaviors for 90 min by using a Microsoft Power Point format. The contents of sleep hygiene included psychoeducation to raise their awareness of their sleep health and to motivate them towards practicing their sleep-promoting behaviors.

**Fig. 1:**
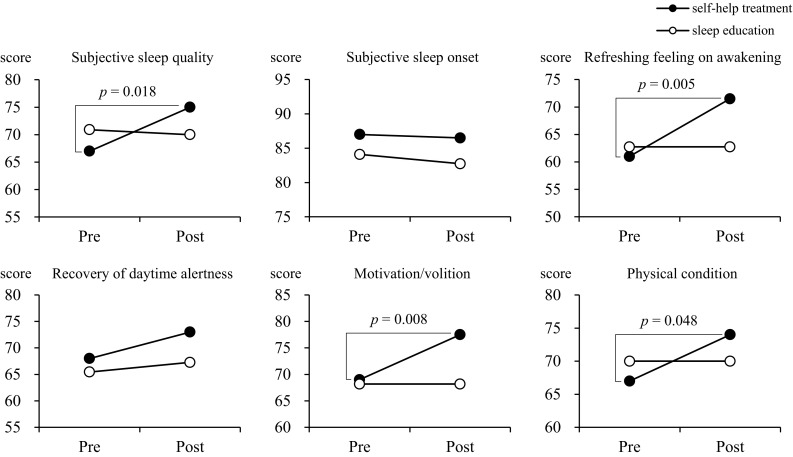
Changes in subjective sleep quality and mental/physical conditions between the self-help treatment and the sleep education groups

Following the sleep education, they were asked to check their own sleep pattern and daytime functioning and to assess sleep-promoting behaviors. They were asked to select three target behaviors as goal setting. Also, the self-help treatment group was asked to record the daily achievement rate using sleep diaries for two weeks. Based on the response (△ but can practice) to checklist (Table 4) of sleep-promoting behaviors, they were asked to both practice and monitor three sleep-promoting behavior sets as their goal for two weeks by using sleep diaries. Sleep diaries were distributed to all the school nurses to provide a subjective measure of sleep and to monitor a goal over the following 14 days. In the self-help treatment group, the bedtime and total sleep time have significantly improved and the irregularity of wake-up time significantly reduced (Fig. 1). Furthermore, the self-help treatment revealed that the lifestyle-habits (getting up in the every morning approximately decided time) showed significant improvements after treatment and improved the sleep, the mood of morning, and the volition significantly. On the other hand, the sleep education improved the lifestyle-habits of “starting with the ability to do, when setting up a target”. Therefore, these findings suggested self-help treatment was effective in the prevention of nocturnal lifestyle and irregularity and promoted an improvement of the sleep, the mood of morning, and the volition.

**Table 4:** Checklist of sleep-promoting behaviors for school teacher and school nurse

Checklist of sleep-promoting behaviors for school teacher and school nurse			
Sleep-promoting behaviors	Self-assessment		
1. Getting up at almost fixed time every morning	○	△	×
2. Having breakfast every morning	○	△	×
3. Exposing oneself to sunlight in the morning	○	△	×
4. Meeting people as much as possible during the day	○	△	×
5. Taking a short nap for 15 to 20 min between 13:00 and 15:00 hours	○	△	×
6. Not taking a nap just after coming back home	○	△	×
7. Exercising for at least 30 min, 2 hrs before bedtime	○	△	×
8. Having dinner 2 hours before bedtime	○	△	×
9. Avoiding caffeinated drinks, such as tea or coffee, after dinner	○	△	×
10. Not going out to brightly lit places, such as convenience store, by 2 hours before bedtime	○	△	×
11. Taking a tepid bath relaxedly	○	△	×
12. Avoiding watching television or using computer for long time	○	△	×
13. Avoiding watching television or reading a book on the bed	○	△	×
14. Not smoking cigarette 1 hour before sleep	○	△	×
15. Making less bright the room 1 hour before sleep	○	△	×
16. Going to bed only after becoming sleepy	○	△	×
17. Keeping the bedroom at comfortable temperature and quiet	○	△	×
18. Trying to rest the brain and mind before sleep	○	△	×
19. Avoiding nightcap liquor	○	△	×
20. Avoiding worrying in the bed	○	△	×
21. Keeping the mobile phone away from bedside before sleep	○	△	×
22. Going to bed by 12:00 midnight at the least every night	○	△	×
23. Keeping a regular sleep time every day	○	△	×
24. Avoiding worries and seeking advice	○	△	×
25. Enjoying hobbies during daytime	○	△	×
26. Trying to start something new that has never before tried	○	△	×
27. Setting a reachable goal and trying to practice	○	△	×
28. Avoiding to have a perfectionist attitude	○	△	×

○ practice, × do not practice, and △ but can practice

### Sleep education with a self-help treatment by teacher for high school students

We examined the effects of Sleep education with a self-help treatment by teacher for high school students [8, 14, 20].

Sixty high school students who gave informed consent participated in the sleep education program using the self-help treatment; the study was carried out for 2 weeks. In this program, self-checking for daily life-habits (Table 5), goal setting for behavioral changes, and self-monitoring were used as behavioral modification techniques. The program ran for 2 weeks in October. A teacher at a high school in Hiroshima lectured the students about sleep hygiene. Next, the teacher asked each student to check their own life-habits and to select three of their behaviors to target. A questionnaire involving lifestyles and sleep health was used to assess the effects of the program. Furthermore, compliance with the target behavioral habits was assessed. After the sleep education using the self-help treatment, the students’ satisfaction of sleep onset (*P* < 0.05), sleep maintenance (*P* < 0.05) significantly improved. Furthermore, the effects of satisfaction of sleep onset and sleep maintenance of students (*n* = 50) sustained beyond the 6 months’ period of the study.

**Table 5:** Sleep knowledge and checklist of sleep-promoting behaviors for high school students

Checklist of sleep-promoting behaviors for students of high school and university			
Sleep-promoting behaviors	Self-assessment		
1. Getting up at almost fixed-time every morning	○	△	×
2. Exposing oneself to sunlight in the morning	○	△	×
3. Having breakfast every morning	○	△	×
4. Meeting people as much as possible during the day	○	△	×
5. Enjoying the club activities and hobbies during the daytime	○	△	×
6. Not taking a nap just after coming back home	○	△	×
7. Avoiding caffeinated drinks, such as tea or coffee, after dinner	○	△	×
8. Having dinner 2 hours before bedtime	○	△	×
9. Not going out to brightly lit places after 9:00 pm	○	△	×
10. Avoiding snacks late in the evening	○	△	×
11. Taking a tepid bath relaxedly	○	△	×
12. Keeping the mobile phone away from bed side before sleep	○	△	×
13. Avoiding to watchign television or reading books on the bed	○	△	×
14. Changing into the night clothes before bedtime	○	△	×
15. Keeping a bedroom at comfortable temperature and quiet	○	△	×
16. Trying to rest the brain and mind before sleep	○	△	×
17. Keeping a regular bedtime every day	○	△	×
18. Going to bed by 12:00 am at the least every night	○	△	×
19. Avoiding worries while in the bed	○	△	×
20. Going to bed only after becoming sleepy	○	△	×
21. Keeping the difference of wake-up time of weekdays and holiday within 2 hour	○	△	×
22. Keeping a regular sleep time every day	○	△	×

○ practice, × do not practice, and △ but can practice

### The effects of short naps and slight exercises in the evening on improving sleep, brain function, physical and mental health for elderly

“Intervention’’ by a short nap after lunch (30 min between 1300 and 1500 h) and exercise with moderate intensity that includes stretching and flexibility in the evening (30 min from 1700 h) have improved sleep quality and mental health [22, 23]. An ‘‘intervention’’ consisting of a short nap and slight exercise in the evening for elderly was carried out for 4 weeks. Their physical activities were recorded using actigraphs for 1-week baseline and post-intervention. After the intervention, wake-time after sleep onset (WASO) significantly decreased, which showed that sleep quality was improved. Nodding in the evening, sleepiness during the daytime, and mental health improved. These studies demonstrated that a proper awakening maintenance during the evening was effective in improving sleep quality and that a modification of lifestyle was also effective. After the intervention, in addition to improvements in sleep, the elderly participants were found to perform significantly better on a computer cognitive task and to have improved brain function [23]. The reduction in daytime drowsiness is thought to have contributed to the improved brain function. Regarding the physical strength and fitness tests, the muscular power of a leg, pliability, and sense of balance significantly increased. Alertness, motivation, physical fatigue, concentration ability, appetite, level of confidence and other parameters also showed significant improvements. After the ‘‘intervention,’’ many elderly stated that their mental and physical health also improved [23].

### Sleep health promotion by assuring proper wakefulness during the day—a sleep-related mini-day service program for mental and physical wellness

New sleep-related mini-day service programs have been proposed [23]. This sleep health promotion problem implements multiple lifestyle changes, including short daytime naps, laughter therapy or group work, and light evening exercise. The long-term effects of maintaining these lifestyle changes to improve sleep. We examined the long-term effects of a sleep-related mini-day service program with 23 elderly people as participating subjects. The subjects of this study were twenty-three elderly people who gave informed consent for their participation. The study program combined short naps (30 min between 13:00 and 15:00), group work (cognitive-behavioral intervention for sleep and stress and positive thinking training), and moderate-intensity exercise, including stretching and flexibility, in the evening (30 min beginning at 17:00). The study was carried out for 4 weeks within a Wellness Program. Sleep was recorded using an actigraph at baseline and 6 months later.

After the intervention, WASO decreased significantly, and mental health and performance of the visual detection task improved. The reduction in daytime drowsiness is thought to have contributed to the improved brain function. The improved sleep and sleep-related lifestyle were sustained beyond the 6 month period of the study.

The results demonstrated that the proper awakening maintenance during the evening was effective in improving sleep quality. These results suggested that health-promoting activities to ensure proper daytime wakefulness and sleep are effective for improving the QOL of elderly people.

### Sleep education with self-help treatment for the local resident

Insomnia is related to hypertension and hyper glycemia (diabetes mellitus), which are closely related to lifestyle. Therefore, lifestyle improvements are the key to solving these issues. Sleep problems become more common with age, affect the quality of life for individuals and their families and caregivers, and can increase healthcare costs. Hypnotics and cognitive behavioral therapy for insomnia (CBT-I) have shown some efficacy. Cognitive and behavioral treatments for sleep problems aim to improve sleep by changing poor sleep habits and challenging negative thoughts attitudes and beliefs about sleep. It has been reported that hypnotics have a risk of adverse events, such as rebound insomnia. CBT, however, requires a large amount of time and effort to change one’s daily practice.

We evaluated improvements in the quality of sleep due to sleep education with self-help treatment in the local residents [8]. Participants were asked to improve their own choose three targets behaviours in a menu of sleep-promoting behaviours for 4 weeks. As the results of sleep education with self-help treatment, significant improvements were observed in the degree of subjective sleep and wake time after sleep onset (WASO). For objective measures by actigraph, sleep onset latency, WASO, and sleep efficiency were improved. GHQ scores (mental Health) also significantly improved. It has been suggested that lifestyle modifications may improve sleep and health. Results of this study suggest that even a minimal improvement in lifestyle is beneficial.

Additionally, we examined whether sleep management with self-help treatment for the elderly promotes the improvement of sleep, QOL, self-efficacy, and blood pressure [24]. The 32 elderly subjects who agreed to participate in our study were provided information about proper sleep hygiene and sleep-promoting behavior, and method to alleviate mental/physical stress symptoms and its exercise. Then they were asked to monitor three sleep-promoting behaviors set as their target behaviors for 2 weeks by using sleep diaries. At post-treatment assessment, a global PSQI score declined from 7.1 to 5.1, and significant improvement was observed in refreshing feeling on awakening, mental/physical QOL, self-efficacy, and maximal blood pressure. Effect sizes in sleep onset latency [0.51 (95 % CI −8.74 to 7.72)] and sleep efficiency [0.42 (95 % CI −3.79 to 4.63)] were moderate, although there were no significant improvements.

When we also examined effective sleep-promoting behaviors for improvements of sleep-related problems or QOL, the stepwise multiple regression analyses revealed that difficulty initiating sleep may improve by practicing getting up at almost fixed-time every day or taking a short nap for 30 min between 13:00 pm and 15:00 pm (Table 6). As for QOL, it is possible that the elderly having a moderate physical activity at an early evening are likely to improve mental health.

**Table 6:** Stepwise multiple regression analyses of the relationships between sleep-promoting behaviors and both sleep-related problems and health-related quality of life

Sleep-promoting behaviors	DIS (≥30 min)		DMS (≥2 times/wk)		Sleep quality		EDS		Physical QOL		Mental QOL		Self-efficacy	
	*β*	*p* value	*β*	*p* value	*β*	*p* value	*β*	*p* value	*β*	*p* value	*β*	*p* value	*β*	*p* value
1. Getting up at almost fixed-time every morning	0.914	<0.001			0.921	<0.001	0.362	0.028					0.605	0.002
2. Having breakfast every morning, chewing well	−0.993	<0.001											−0.271	0.042
3. Exposing oneself to sunlight in the morning			−0.739	<0.001	0.396	0.005								
4. Meeting people as much as possible during the day	0.250	0.012							0.470	0.015			0.557	0.007
5. Walking a lot during the daytime														
6. Enjoying the hobbies during the daytime														
7. Exposing yourself to sunlight in the afternoon			0.604	<0.001	0.304	0.011	0.369	0.019						
8. Taking a short nap for 30 min between 13:00 and 15:00 (over 55 yrs old)	0.733	<0.001												
9. Having a moderate physical activity, such as walking, at an early evening											0.329	0.096	0.704	0.003
10. Not taking a nap just after coming back home			0.384	0.014										
11. Avoiding caffeinated drinks, such as tea or coffee, after an early evening					−0.851	<0.001							0.377	0.025
12. Not smoking cigarette 1 hour before sleep														
13. Keeping the bedroom dimly lit 1 hour before sleep														
14. Taking a tepid bath relaxedly														
15. Avoiding watching television or reading a book on the bed													0.282	0.057
16. Keeping the bedroom at comfortable temperature and quiet					−0.776	0.001								
17. Trying to rest the brain and mind before sleep			−0.255	0.075										
18. Avoiding nightcap liquor							−0.348	0.051			−0.464	0.023		
19. Avoiding worrying in the bed	0.369	0.009			−0.383	0.005								
20. Going to bed only after becoming sleepy					−0.494	0.003	−0.704	0.007						
21. Keeping a sleep time every day at the length appropriate to oneself	0.258	0.005			0.444	0.002								
22. Keeping a regular sleep time every day	0.957	<0.001	0.613	<0.001										
23. Taking a short nap for 15 to 20 min between 13:00 and 15:00(under 55 yrs old)														
*R* (*R* ^2^)	0.980 (0.960)		0.942 (0.887)		0.973 (0.947)		0.736 (0.541)		0.470 (0.221)		0.487 (0.238)		0.977 (0.954)	
*p* value	<0.001		<0.001		<0.001		0.004		0.020		0.044		0.003	

*DIS* difficulty initiating sleep, *DMS* difficulty maintaining sleep, *EDS* excessive daytime sleepiness, *QOL* quality of life. Sleep quality, EDS, physical/mental QOL and self-esteem were measured with the Pittsburgh Sleep Quality Index (PSQI), the Epworth Sleepiness Scale, the Short Form-8 and the General Self-Efficacy Scale, respectively; DIS and DMS were assessed with subordinate categories in PSQI

Hence, we recommend the sleep management with self-help treatment (monitoring sleep-promoting behaviors set as target) as a valid treatment option to improve elderly sleep problems, self-efficacy, and health-related QOL.

## Conclusion

Present results suggest sleep education using a short-term cognitive-behavioral method (knowledge of sleep and self-help treatment) improves the quality of sleep, arousal levels, daytime concentration, and motivation. We referred to the importance of sleep education and sleep management in schools and suggest that sleep improvement assistance requires (1) the dissemination of appropriate knowledge, (2) providing support tools, and (3) the development of human resources. Moreover, these findings strongly suggest that reconsidering lifestyles and ensuring high-quality sleep will be effective in greatly reducing the number of elderly with dementia or confined to bed. The number of such elderly is expected to dramatically increase in the future. Comfortable sleep in old age will not only result in a clear increase in the QOL of elderly people themselves but will also be important for an increased well-being of the family and caregivers of the elderly, as well as society as a whole.
